# *Phaeobacter gallaeciensis* Reduces *Vibrio anguillarum* in Cultures of Microalgae and Rotifers, and Prevents Vibriosis in Cod Larvae

**DOI:** 10.1371/journal.pone.0043996

**Published:** 2012-08-22

**Authors:** Paul W. D’Alvise, Siril Lillebø, Maria J. Prol-Garcia, Heidrun I. Wergeland, Kristian F. Nielsen, Øivind Bergh, Lone Gram

**Affiliations:** 1 National Food Institute, Technical University of Denmark, Lyngby, Denmark; 2 Department of Biology, University of Bergen, Bergen, Norway; 3 Department of Systems Biology, Center for Microbiological Biotechnology, Technical University of Denmark, Lyngby, Denmark; 4 Institute of Marine Research, Bergen, Norway; The University of Plymouth, United Kingdom

## Abstract

*Phaeobacter gallaeciensis* can antagonize fish-pathogenic bacteria *in vitro*, and the purpose of this study was to evaluate the organism as a probiont for marine fish larvae and their feed cultures. An *in vivo* mechanism of action of the antagonistic probiotic bacterium is suggested using a non-antagonistic mutant. *P. gallaeciensis* was readily established in axenic cultures of the two microalgae *Tetraselmis suecica* and *Nannochloropsis oculata*, and of the rotifer *Brachionus plicatilis*. *P. gallaeciensis* reached densities of 10^7^ cfu/ml and did not adversely affect growth of algae or rotifers. *Vibrio anguillarum* was significantly reduced by wild-type *P. gallaeciensis*, when introduced into these cultures. A *P. gallaeciensis* mutant that did not produce the antibacterial compound tropodithietic acid (TDA) did not reduce *V. anguillarum* numbers, suggesting that production of the antibacterial compound is important for the antagonistic properties of *P. gallaeciensis*. The ability of *P. gallaeciensis* to protect fish larvae from vibriosis was determined in a bath challenge experiment using a multidish system with 1 larva per well. Unchallenged larvae reached 40% accumulated mortality which increased to 100% when infected with *V. anguillarum*. *P. gallaeciensis* reduced the mortality of challenged cod larvae (*Gadus morhua*) to 10%, significantly below the levels of both the challenged and the unchallenged larvae. The TDA mutant reduced mortality of the cod larvae in some of the replicates, although to a much lesser extent than the wild type. It is concluded that *P. gallaeciensis* is a promising probiont in marine larviculture and that TDA production likely contributes to its probiotic effect.

## Introduction

One of the major challenges of marine aquaculture is the continuous and reliable production of juveniles. Severe losses in marine larviculture are caused by infection with opportunistic pathogenic bacteria, including several members of the *Vibrionaceae* family [1], [2], that accounts for approximately 1.5% of the bacterial community in the oceans [3]. Only some *Vibrio* species are pathogenic to organisms reared in marine aquaculture and one of the most prominent fish and shellfish pathogens is *Vibrio (Listonella) anguillarum* that causes serious disease and mortalities [2]. The main source of pathogenic bacteria in marine aquaculture is supply water [4], but also brood stock, humans, or starter cultures are possible sources of pathogens [5]. The majority of marine fish larvae are reared intensively in presence of microalgae (green water), which improves feeding, growth, and survival of the larvae [6]–[9]. The larvae require live feed, and rotifers (*Brachionus plicatilis*) are typically used as first feed. The rotifers themselves are fed or enriched with live microalgae, such as *Tetraselmis suecica*, *Nannochloropsis oculata*, and *Isochrysis galbana*. Opportunistic pathogens can proliferate in larval feed cultures of phytoplankton and invertebrates due to high concentrations of organic matter. Algae, rotifer and *Artemia* cultures can therefore harbor high concentrations of pathogenic bacteria [1], [5], [10], [11]. Prophylactic treatment of larvae or their feed cultures with antibiotics can reduce the pathogen load, but has to be avoided, since it leads to emergence of antibiotic-resistant pathogens, and since it impedes the establishment of a normal non-pathogenic microbiota [1], [12], [13].

The potential use of probiotic bacteria to limit outbreaks or effects of bacterial diseases in fish and invertebrate cultures has been investigated for more than two decades. Most studies have focused on the intestinal microbiota [14]–[19], although the use of probiotics is not confined to the intestinal tract of the cultured organisms [15], [20]. Biotic and abiotic surfaces, algal and fecal particles, and the nutrient-rich water serve as habitat and reservoir of opportunistic pathogenic bacteria in cultures of fish larvae or their food organisms [1], [5], [10], [11], [20], and it is hypothesized that competition by non-pathogenic bacteria that are superior in colonizing and persisting in these habitats could reduce the incidence of pathogenic bacteria.

*Phaeobacter gallaeciensis* (formerly *Roseobacter gallaeciensis*) is a Gram-negative *α-*proteobacterium from the *Roseobacter*-clade [21]. The bacterium produces the antibacterial compound tropodithietic acid (TDA) that is an efficient inhibitor of *V. anguillarum* and other fish-pathogenic bacteria [22]–[25]. *Phaeobacter* spp. are commonly isolated from larval cultures of marine fish and shellfish [26]–[28], and do not appear to adversely affect fish larvae [22], [29]. *Ruegeria mobilis*, a close relative to *Phaeobacter* also producing TDA, is a cosmopolitan marine bacterium that can be isolated from most ocean waters, apart from polar waters [30].

In a previous study, it was demonstrated that *Phaeobacter* and *Ruegeria* isolated from a turbot hatchery [26] could eliminate *V. anguillarum* in a seawater-based combined liquid-surface system [22]. It was demonstrated, using a TDA-negative mutant, that TDA production was likely a key factor in the pathogen inhibition. The purpose of the present study is to determine if *P. gallaeciensis* BS107 (DSM 17395) could antagonize *V. anguillarum* in fish larvae and cultures of their feed organisms. To the authors' knowledge, no study on antagonistic probiotic bacteria has yet elucidated the mechanism of action *in vivo*. Therefore a non-antagonistic TDA-negative mutant of *P. gallaeciensis* BS107 (DSM 17395) was created to investigate the *in vivo* mechanism of action, as emphasized by Tinh *et al*. [15]. The type strain *P. gallaeciensis* BS107 (DSM17395) [28] was chosen, since its inhibition of *V. anguillarum* in *Tetraselmis* cultures was more pronounced than that of other *Phaeobacter* and *Ruegeria* strains, as assessed in a preliminary experiment (data not shown). Gnotobiotic algae and rotifers were used for studying probiotic and nutritional effects of the introduced organism, as recommended [15], [31], [32].

## Materials and Methods

### Bacterial strains and media

All strains and plasmids are listed in Table 1. *Phaeobacter (Roseobacter) gallaeciensis* BS107 (DSM17395) was isolated from seawater in scallop (*Pecten maximus*) cultures [28]. *Vibrio anguillarum* serotype O1 strain NB10 was used in algal and rotifer experiments. It was isolated from the Gulf of Bothnia and has caused disease in rainbow trout [33], [34]. The strain has been tagged by insertion of plasmid pNQFlaC4-gfp27 (*cat*, *gfp*) into an intergenic region on the chromosome, and was kindly provided by D. Milton, University of Umeå [35]. *V. anguillarum* serotype O2α HI610 was used in challenge trials with cod larvae. The strain was isolated from diseased cod juveniles in the closed seawater basin Lake Parisvatn by the Institute of Marine Research (IMR), Norway, and has been used in challenge trials with cod [36]–[39]. It has been selected from a group of *V. anguillarum* strains of different serotypes, being the strain that caused the highest mortality in challenge trials with turbot, halibut and cod larvae [40].

**Table 1:** Bacterial strains and plasmids.

Strain or plasmid	Genotype or relevant markers	Source or reference
**Strains**		
*P. gallaeciensis* BS107 (DSM17395)	Wild type	Ruiz-Ponte *et al.* 1998 [28]
*P. gallaeciensis* BS107-Pda8	CDS104961::EZ-Tn5, Kan^R^	This study
*P. gallaeciensis dsRed*	MiniTn7(Gm^R^)P_A1/04/03_ *DsRed*Express-a	This study
*V. anguillarum* NB10	Serotype O1, cm^R^, PA1/04/03-RBSII-*gfp*mut3*-T1	Croxatto *et al.* 2007 [35]
*V. anguillarum* HI610	Serotype O2α	Samuelsen & Bergh 2004 [36]
**Plasmids**		

Bacteria from frozen stock cultures (−80°C) were streaked on half-strength Marine Agar (½MA; 27.6 g Difco 212185 Marine Agar, 15 g Instant Ocean Sea Salts, 7.5 g Agar, 1 l deionized water). ½MA was also used for counting *P. gallaeciensis*. *V. anguillarum* was counted on Tryptone-Soy Agar (TSA; Oxoid CM0131) containing 6 mg/l chloramphenicol. The bacterial precultures for the algae and rotifer experiments were grown in 20 ml of ½YTSS (2 g Bacto Yeast extract, 1.25 g Bacto Tryptone, 20 g Sigma Sea Salts, 1 l deionized water) [41] at 25°C with aeration (200 rpm) until OD_600_ = 1.0. The cells were harvested at 5,000 x *g*, washed twice, and used as inoculum for algae and rotifer experiments. Bacteria were diluted and washed in artificial seawater (ASW; 2% Sigma Sea Salts). Axenicity of algae and rotifer cultures was controlled by plating 100 µl on ½MA and incubating for 7 days at 25°C.

For the challenge trials, *V. anguillarum* HI610 was grown in tryptone-soy broth with additional 0.5% NaCl at 20°C with shaking at 60 rpm to an OD_600_ of about 0.5. The *P. gallaeciensis* strains were grown in MB without shaking at 20°C until stationary phase was reached. All strains were harvested by centrifugation (1,825 x *g*), washed twice, and resuspended in aerated autoclaved 80% seawater. The bacterial concentrations in these suspensions were determined using a counting chamber for *V. anguillarum*, and for the *P. gallaeciensis* strains by measuring OD_600_ after centrifugation and dissolving in 0.1M NaOH.

### Generation of a TDA-negative *Phaeobacter* mutant

A mutant library of *P. gallaeciensis BS107* was created by random transposon insertion mutagenesis using the EZ-Tn5 <R6Kγori/KAN>Tnp Transposome Kit (Epicentre, Madison, WI), following the procedure of Geng *et al*. [42]. Ten non-pigmented mutants were selected, and absence of TDA production was confirmed by UHPLC-TOFMS and in an agar-diffusion test against *V. anguillarum* [27]. Growth rates of selected mutants were compared to the wild type in aerated (200 rpm) ½YTSS cultures at 30°C, and one of the strains with a growth rate comparable to the wild type, BS107-Pda8, was chosen for further experiments. Using rescue cloning as described in the transposome kit manual, the mutated locus was identified as CDS104961, which encodes for a “periplasmic component of a TRAP-type C4-dicarboxylate transport system”, as annotated on the BS107 genome on www.roseobase.org.

### Fluorescence tagging of *Phaeobacter*

*P. gallaeciensis* BS107 was tagged chromosomally with a miniTn7(Gm)P_A1/04/03_ *DsRedExpress-a* cassette, using a mini-Tn7 tagging system [43], [44]. The delivery and helper plasmids were electroporated into *P. gallaeciensis*, followed by selection on ½MA containing 75 µg/ml gentamicin. pPDA11, a transcriptional fusion of the *tdaC* promoter to a promoterless *gfp* gene ligated to the broad-host range plasmid pRK415, was constructed in an analogous manner to pHG1011 as described in Geng *et al*. 2010 [45].

### *Phaeobacter* antagonism in algae

*Tetraselmis suecica* CCAP 66/4 (*Prasinophyceae*) was obtained as axenic culture from the Culture Collection of Algae and Protozoa (Oban, UK). It was cultured in B-medium [46], a mineral algae medium, based on ASW. The 250-ml culture bottles were closed with cotton plugs and slowly aerated through a 0.2 µm syringe filter and a silicone tube, to prevent settling of particles. Light intensity on the bottles was 13,000 lux (daylight spectrum). Algal concentrations were assessed by measuring absorption at 665 nm, and calibrating with counts of axenic reference cultures in a Neubauer-improved counting chamber, using formaldehyde as fixative (0.5% final concentration). For each *V. anguillarum* inoculum level tested, eight bottles of 150 ml of B-medium were inoculated with 6.6×10^4^ cells/ml axenic *T. suecica*. Two bottles were inoculated with approximately 10^7^ cfu/ml washed *P. gallaeciensis* BS107, two bottles with the same level of washed mutant *P. gallaeciensis* BS107-Pda8 cells, and four bottles were left axenic. The cultures were grown for 2 days and axenicity was checked. All cultures, except two axenic negative controls, were inoculated with *V. anguillarum* NB10 to concentrations of 10, 100, 1000, or 10^4^ cfu/ml. Inoculum levels were verified by plate-counting. Concentrations of algae and both bacterial species were observed until day 5 after inoculation of the pathogen. Two independent experiments were performed for every initial concentration of *V. anguillarum*. ***Nannochloropsis oculata*** CCMP525 (*Eustigmatophyceae*) was obtained as axenic culture from the Center for Culture of Marine Phytoplankton (West Boothbay Harbor, ME). Since it did not grow in ASW-based B-medium, it was cultured in f/2-medium [47] based on Atlantic Seawater obtained from CCMP. *N. oculata* cultures were not aerated. Antagonism experiments were done as in *T. suecica*, but only one initial *V. anguillarum* concentration (10^4^ cfu/ml) was tested. Two independent experiments with two different initial densities of algae (lower density: 4×10^6^ cells/ml, higher density: 2×10^7^ cells/ml) were carried out.

### TDA analysis

Samples of *T. suecica – P. gallaeciensis* co-cultures (20 ml) were extracted in 50-ml falcon tubes with 30 ml ethyl acetate (HPLC grade) containing 1% formic acid (HPLC grade) on a shaking table for 1 h. The samples were centrifuged at 8,000 x *g*, and 26 ml of the upper phase was transferred to a new Falcon tube and evaporated to dryness at 35°C with nitrogen flow. The samples were resuspended in 300 µl 85% acetonitrile, vortexed for 5 sec, placed in an ultrasonication bath for 10 min, vortexed again for 5 sec and filtered through a standard 0.22 µm PFTE syringe filter into a HPLC vial. Subsamples of 2 µl were then analyzed by UHPLC-TOFMS on a maXis G3 quadrupole time of flight mass spectrometer (Bruker Daltonics, Bremen, Germany) equipped with an electrospray (ESI) ion source which was connected to an Ultimate 3000 UHPLC system (Dionex, Sunnyvale, CA). Separation was performed at 40°C on a 100 mm×2.1 mm ID, 2.6 µm Kinetex C_18_ column (Phenomenex, Torrance, CA) equipped with Kinetex pre-column using a water-acetonitrile gradient (both buffered with 20 mM formic acid) at a flow of 0.4 ml min^−1^ starting at 10% acetonitrile and increased to 100% in 10 min, keeping this for 3 min. MS was operated in ESI^+^ with a data acquisition range of m/z 100–1000 at a resolution of 40,000 FMWH, the MS was calibrated using 20 mM sodium formate infused prior to each analytical run, providing a mass accuracy better than 1.5 ppm. TDA was detected and quantified from the extracted ion chromatograms of the [M+H]^+^ ions (± m/z 0.001).

For quantification, *T. suecica* cultures were spiked to a final concentration of 4800, 2400, 1600, 800, 320, 160, and 0 (blank) nM TDA (BioViotica, Dransfeld, Germany) by adding a maximum of 80 µl TDA-acetonitrile solution, and treated as described above. Spiked samples were left at room temperature for at least 1 h prior to extraction. The method was validated on 3 different days using spiked samples as described, and no false positives or negatives were recorded. Relative standard deviation was 30% and the limit of detection was estimated to be <50 nM (signal/noise 5∶1), based on the blank samples and lower calibration points. Sensitivity was greatly influenced by the age of the UHPLC column since TDA tailed (although a new pre-column was used) on columns which had been in use for only a few weeks. Samples from two individual biological experiments were analyzed independently.

Absence of TDA in static cultures of the TDA-negative mutant BS107-Pda8 was confirmed by UHPLC-TOFMS analysis.

### Phaeobacter antagonism in rotifers, Brachionus plicatilis

A stock of the rotifer *B. plicatilis* (L-type) was obtained from Reed Mariculture (Campbell, CA). Axenic rotifers were attained by disinfecting approximately 50 amictic rotifer eggs in 1 ml of a strong antibiotic solution (150 µg/ml Tetracycline, 300 µg/ml Kanamycin, 60 µg/ml chloramphenicol, 1000 U/ml Penicillin in ASW) for 2 days. The hatched rotifers were filtered onto a sterile 50-µm polyamide mesh, rinsed with ASW and from then on fed with concentrated axenic *T. suecica*. No experiment with *N. oculata* as rotifer feed was conducted, since survival of *V. anguillarum* in presence of *N. oculata* was very variable (see Results section). Rotifer densities were determined by counting in a Sedgewick-Rafter counting chamber. Before counting, the cultures were thoroughly mixed, and 100 µl 5% formaldehyde added to a 1 ml sample. To set up co-culture experiments, axenic rotifer cultures were divided into eight 20-ml batches in 50-ml centrifuge tubes. The initial rotifer concentrations were 94 /ml (first replicate) and 30 /ml (second replicate). For each replicate, duplicate cultures were inoculated with washed wild type and mutant *P. gallaeciensis* at approximately 5×10^7^ cfu/ml. On the next day, all cultures except the axenic controls were inoculated with 10^4^ cfu/ml *V. anguillarum*. All rotifer cultures were fed daily with 10-fold concentrated *T. suecica* (1–2 ml depending on average rotifer density), so that the algae concentration was at approximately 10^6^ cells/ml after feeding and did not drop below 2×10^5^ cells/ml. The rotifers were counted daily, concentrations of *V. anguillarum* and *P. gallaeciensis* were determined, and axenicity of the negative controls was checked. The rotifer culture samples for enumeration of bacteria were homogenized by grinding and repeated pipetting through a 100-µl pipette tip. This was compared to homogenization with an Ultra-Turrax T25 (IKA, Germany) at 16,000 rpm and no significant differences in bacterial counts were found (P = 0.74).

### Challenge trial

The protocol was adapted from [39], [40]. Cod (*Gadus morhua*) embryos were obtained from the commercial hatchery Havlandet AS, in Florø, Western Norway. Transport of the embryos in polystyrene containers at around 8°C took 4 to 5 hours in total by boat and car. Two independent replicates of the challenge trial were conducted. The embryos used in the first trial were disinfected with Buffodine (Evans Vanodine, Preston, UK), the embryos for the second trial were left untreated. Upon arrival, the embryos were randomly picked and distributed to the wells of 24-well dishes (Nunc, Roskilde, Denmark) filled with 2 ml 80% autoclaved, aerated seawater, placing one embryo in each well. In each trial three dishes for each treatment (72 embryos) were prepared and inoculated immediately. The six treatment groups are listed in Table 2. All inocula were prepared in a volume of 100 µl, and the strains were not mixed before inoculation. Initial bacterial concentrations were 1×10^6^ cfu/ml for *V. anguillarum* HI610 and about 10^7^ cfu/ml for the *P. gallaeciensis* strains. The plates were incubated in the dark at 7°C. The day when 50% of the larvae had hatched was defined as day 0, which was 6 days after the start of the experiment. Dead larvae were registered daily for 14 days.

**Table 2:** Group numbers and treatments in the challenge trial.

Group number	Treatment
T1	Negative control; no bacteria added
T2	Positive control; *V. anguillarum* O2α HI610 10^6^ cfu/ml
T3	Wild type *P. gallaeciensis* BS107 (DSM17395) ∼10^7^ cfu/ml
T4	TDA-mutant *P. gallaeciensis* BS107-Pda8 ∼10^7^ cfu/ml
T5	*V. anguillarum* O2α HI610 10^6^ cfu/ml and wild type *P. gallaeciensis* BS107 (DSM17395) ∼10^7^ cfu/ml
T6	*V. anguillarum* O2α HI610 10^6^ cfu/ml and TDA-mutant *P. gallaeciensis* BS107-Pda8 ∼10^7^ cfu/ml

### Statistics

Differences between concentrations of bacteria or algae were assessed using repeated measures ANOVA after log-transformation. Tukey's multiple comparison test was used for pairwise comparisons. To address the effects of *P. gallaeciensis* presence on concentrations of *V. anguillarum*, initial values (day 0) were omitted in the analysis and the experiments were analyzed separately. Rotifer numbers were not log-transformed before applying ANOVA, and initial values were omitted. Numbers of *P. gallaeciensis* and homogenization methods were compared using paired t-tests after log-transformation.

The cumulative mortalities in the challenge trials were compared at day 10, prior to the onset of starvation towards the end of the experiment. A chi-square test for 2^2^ contingency tables was implemented, using the software R, version 2.13.1 (R Foundation for Statistical Computing, Vienna, Austria).

## Results

### Antagonism in algae cultures

**Figure 1:**
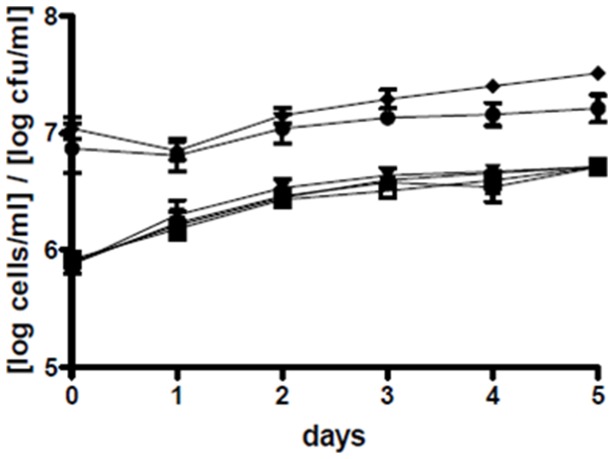
Concentrations of *Tetraselmis suecica* and *Phaeobacter gallaeciensis* in the co-cultures. Means and standard deviations of eight experiments: colony-forming units of *P. gallaeciensis* wild type (♦) and the TDA-negative mutant (•), and concentrations of *T. suecica* with *V. anguillarum* (▾), *T. suecica* with *P. gallaeciensis* wild type (▪), *T. suecica* with *P. gallaeciensis* TDA-negative mutant (▴), and axenic *T. suecica* (□). The *P. gallaeciensis* strains were inoculated at 10^7^ cfu/ml and remained as a steady population, while the algae went from late log into stationary phase.

**Figure 2:**
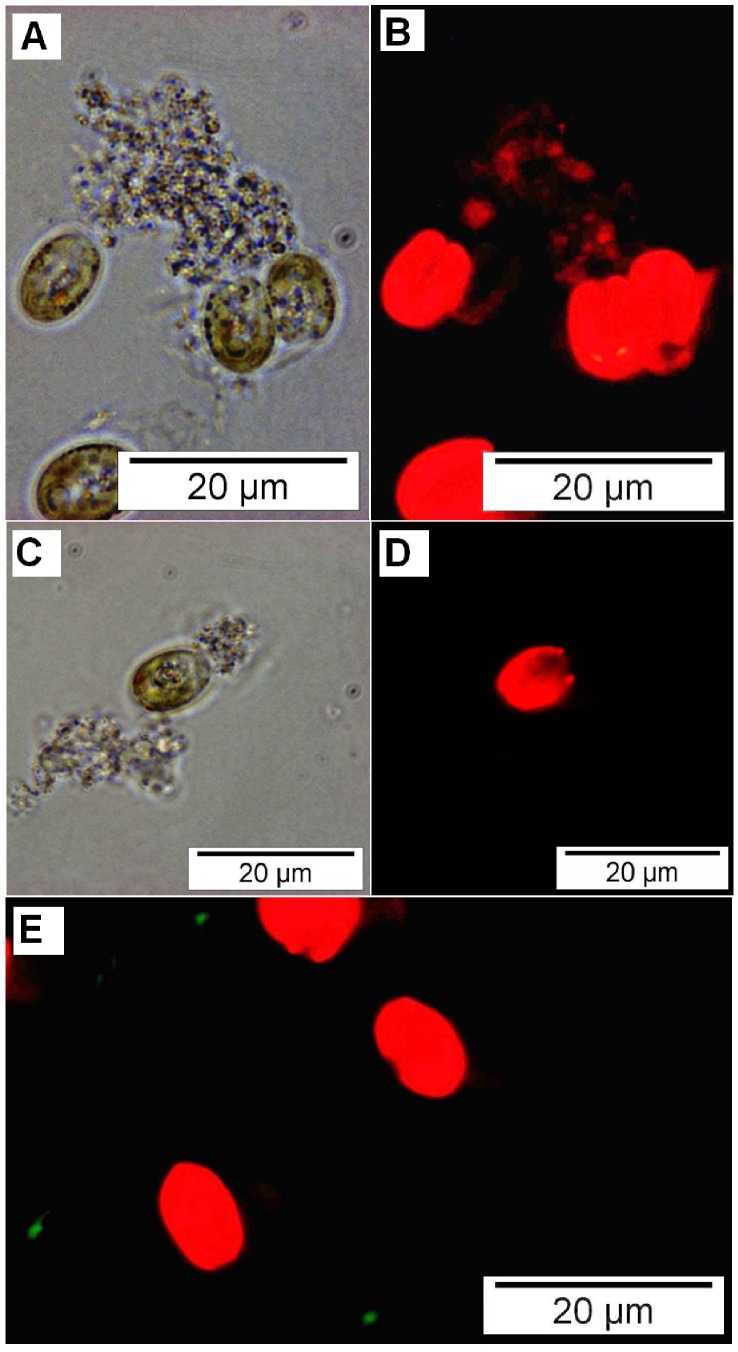
Localization of bacteria in cultures of *Tetraselmis suecica*. Phase-contrast (A,C) and fluorescence (B,D,E) micrographs. Co-culture of *Tetraselmis suecica* with *Phaeobacter gallaeciensis dsRed* (A,B), axenic *T. suecica* (C,D), co-culture of *T. suecica* with *V. anguillarum gfp* (E). Panel A and B show two single (left) and one dividing algal cell (right side), and a marine snow-like particle consisting of algae-debris which is colonized by red-fluorescent *P. gallaeciensis*. Red fluorescence of algae is due to chlorophyll. Panels C and D show an algal cell and particles from an axenic culture, recorded using the same settings as for the panels above. Panel E shows red-fluorescent algae cells and green-fluorescent *V. anguillarum*, which do not colonize particles, but remain in suspension as single, motile cells.

Both wild type and mutant *P. gallaeciensis* colonized the cultures of *T. suecica* and *N. oculata*. In *T. suecica* cultures, *P. gallaeciensis* reached 10^7^ cfu/ml (Figure 1). In the dense cultures of *N. oculata*, *P. gallaeciensis* concentrations were approximately 5×10^6^ cfu/ml, and in the less dense cultures approximately 8×10^5^ cfu/ml (Figure S1). The wild type *Phaeobacter* reached slightly higher numbers in *T. suecica* than the TDA-negative mutant (P = 0.0211). This same slight difference was seen in one of the two *Nannochloropsis* experiments (P = 0.0335, P = 0.9259). *P. gallaeciensis* did not affect growth of the algae *T. suecica* (P = 0.9977) and *N. oculata* (P = 0.9919). Particles consisting of dead *T. suecica* and algal cell walls that were shed during cell division served as habitat for rosette forming *P. gallaeciensis* that formed dense biofilms on the particles (Figure 2 A–D).

**Figure 3:**
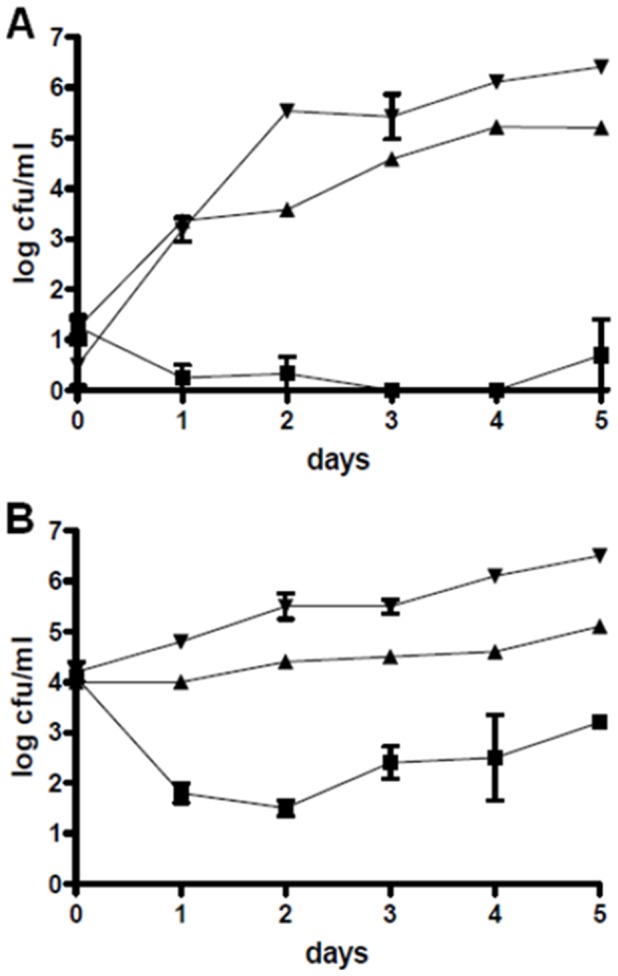
Reduction of *V. anguillarum* in cultures of *Tetraselmis suecica* by *Phaeobacter gallaeciensis*. Colony-forming units of *V. anguillarum* inoculated at 10^1^ cfu/ml (A) and at 10^4^ cfu/ml (B) in presence of *P. gallaeciensis* wild type (▪), in presence of the *P. gallaeciensis* TDA-negative mutant (▴), and in the monoxenic control (▾).

*V. anguillarum* effectively colonized all *Tetraselmis* cultures that were not inoculated with *P. gallaeciensis* and numbers increased by up to 2.7 log units within the first day (Figure 3, Figure S2) and reached an average of 3×10^6^ cfu/ml after 5 days. *V. anguillarum* did not colonize particles in the algae cultures, but remained in suspension (Figure 2 E). The numbers of *V. anguillarum* decreased markedly in presence of wild type *P. gallaeciensis* (Figure 3, Figure S2). *Vibrio* reductions were in the order of 3 log units, as compared to the monoxenic controls with only *V. anguillarum*, and complete elimination of the lowest *Vibrio* inoculum was achieved in 3 out of 4 replicates (Figure 3A). The effects of wild type *P. gallaeciensis* on *V. anguillarum* were highly significant in all *Tetraselmis* experiments, as compared to the controls (P<0.001) and to the mutant (P<0.001). Presence of the TDA-negative mutant did decrease concentrations of the pathogen by about one log unit, although this was only significant (α = 0.05) for two of the initial *Vibrio* concentrations (10^1^: P = 0.0518, 10^2^: P = 0.0011, 10^3^: P = 0.0517, 10^4^: P = 0.0008).

**Figure 4:**
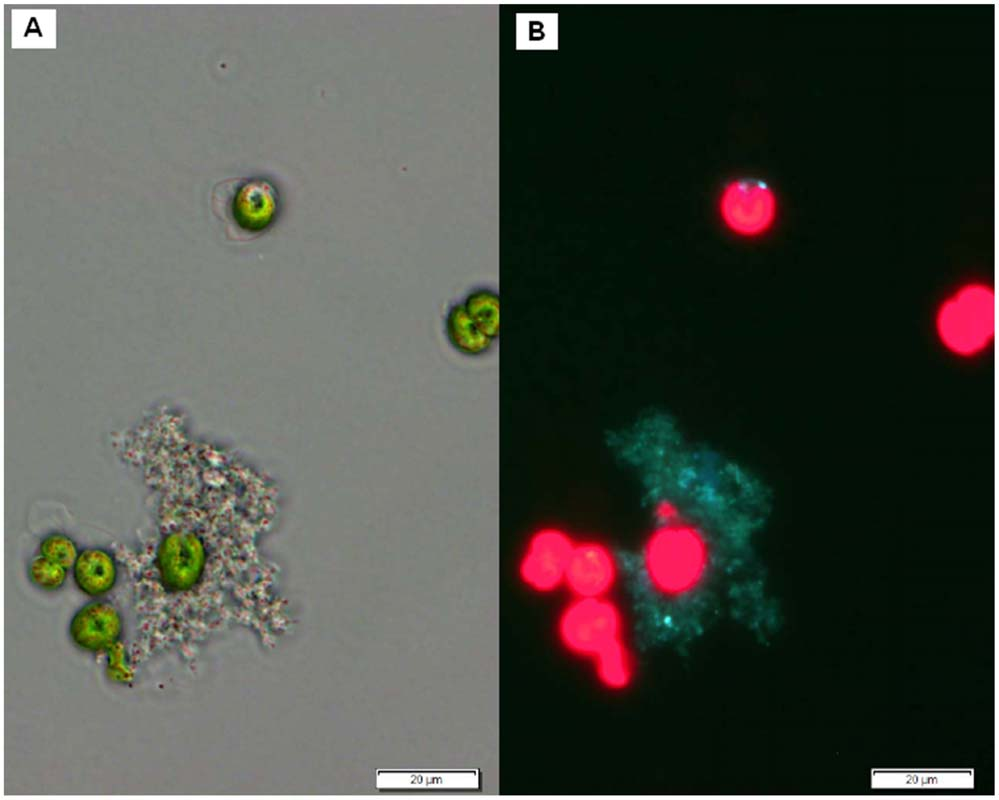
Expression of *tdaC* in co-culture with *Tetraselmis suecica*. Phase contrast (A) and fluorescence (B) micrographs of *P. gallaeciensis* pPDA11 (*tdaCp::gfp*) in co-culture with *T. suecica*. The two panels show the same seven algal cells of which some are dividing, and a marine snow-like particle which is colonized by *P. gallaeciensis* carrying the promoter-fusion on a plasmid. The green fluorescence of *P. gallaeciensis* on the particle shows that the gfp gene is expressed from the tdaC promoter, indicating production of TDA.

The marked difference in *V. anguillarum* inhibition by the wild type *P. gallaeciensis* and the TDA negative mutant suggested that TDA was a major effector molecule. However, TDA was not detected by chemical analysis of the *Phaeobacter–Tetraselmis* co-cultures, where triplicate cultures were each analyzed in triplicates with a detection limit <50 nM TDA. The experiment and analysis were repeated with the same result. To determine if the wild type did indeed produce TDA in the algal cultures, a *P. gallaeciensis* carrying pPDA11 (*tdaCp::gfp*) was co-cultured with *T*. suecica. The *tdaC* promoter, indicative of TDA production, was induced when growing on particles in a *T. suecica* culture, as indicated by Gfp fluorescence (Figure 4). Adding pure TDA to *Tetraselmis* cultures inoculated with *V. anguillarum* caused a complete killing of the *Vibrio* population, but also affected survival of algae (50 µM TDA). A 50-fold lower concentration (1 µM) had no effect on the algae, but temporarily reduced *V. anguillarum* below 10 cfu/ml. A concentration of 50 nM TDA, which was the detection limit of the chemical analysis, did not have any effect (data not shown).

*V. anguillarum* was completely eliminated in *Nannochloropsis oculata* cultures by wild type *P. gallaeciensis* within one or two days (Figure S3). However, *V. anguillarum* could only persist in dense cultures of *N. oculata*. In less dense *N. oculata* cultures *V. anguillarum* disappeared from the monoxenic control within 3 days. Consequently, the effect of the wild type *P. gallaeciensis* on *V. anguillarum*, as compared to the control, was significant in the experiment with high algae density (P = 0.001), but not in low density (P = 0.2106).

### Antagonism in rotifer cultures

**Figure 5:**
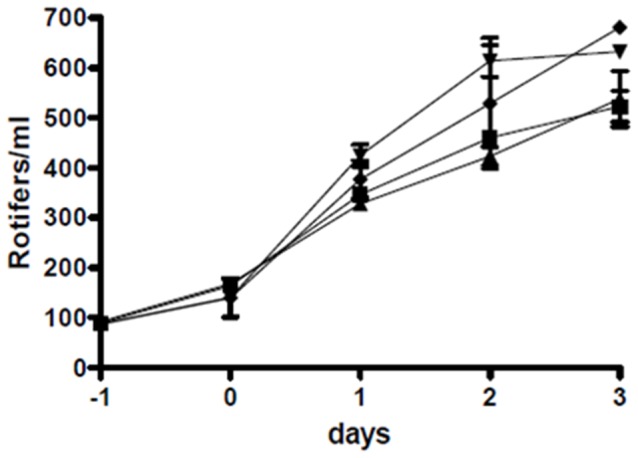
Influence of bacterial strains on rotifer growth. Rotifer numbers in co-culture with *P. gallaeciensis* wild type (▾), with the TDA-negative mutant of *P. gallaeciensis* (♦), with only *V. anguillarum* (▴), and axenic rotifers (▪), first experiment. All bacteria were inoculated at day 0. Both *P. gallaeciensis* strains promoted rotifer growth, whereas *V. anguillarum* had no influence.

**Figure 6:**
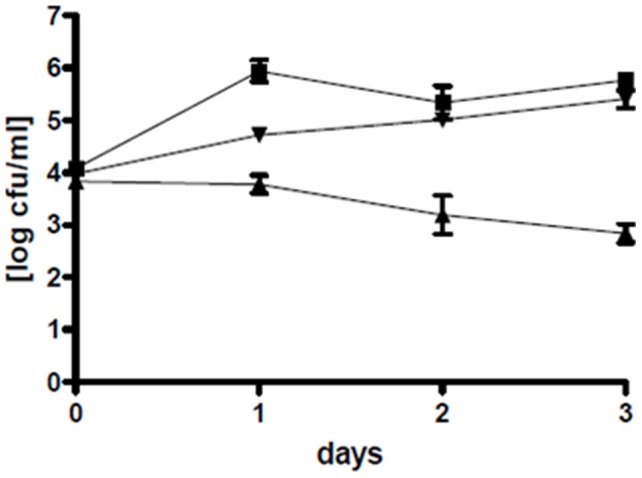
Reduction of *Vibrio anguillarum* by *Phaeobacter gallaeciensis* in rotifer cultures. Mean values of two duplicate experiments: colony-forming units of *V. anguillarum* in co-culture with *P. gallaeciensis* wild type (▴), with the TDA-negative mutant of *P. gallaeciensis* (▾), and in the monoxenic control (▪).

The concentrations of *P. gallaeciensis* and its mutant in the rotifer cultures were stable at about 10^6^–10^7^ cfu/ml, and no significant difference between the two strains was observed (P = 0.3689). The rotifers grew faster and reached higher densities in presence of *P. gallaeciensis* than in the axenic or monoxenic (*V. anguillarum*) controls (P<0.05) (Figure 5, Figure S4). Wild type *P. gallaeciensis* reduced *V. anguillarum* concentrations by 3 log units (P<0.01), in average from 6×10^5^ to 9×10^2^ cfu/ml (Figure 6). The effect of the TDA-negative mutant on the concentration of the pathogen was not significant (P>0.05).

### Challenge trial

**Figure 7:**
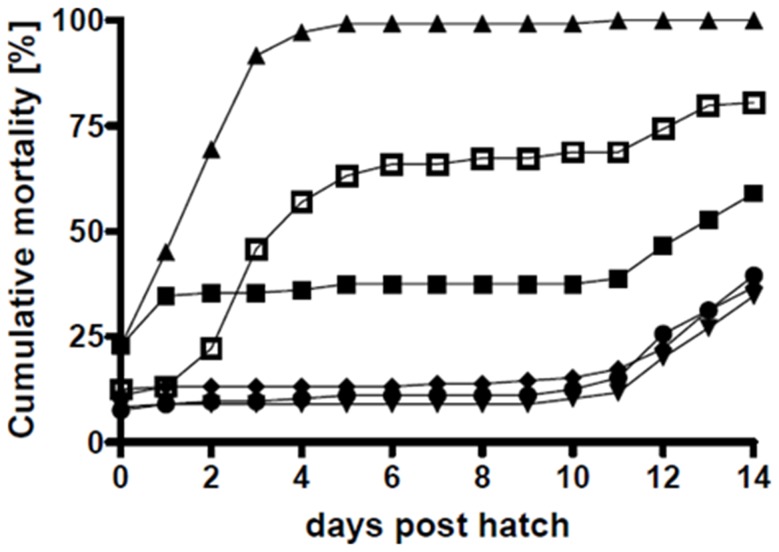
Mortality of cod larvae during the challenge trials. Mean values of two independent triplicate experiments. The single-larvae cultures were simultaneously inoculated with *P. gallaeciensis* wild type and *V. anguillarum* (T5, •), or with the TDA-negative mutant of *P. gallaeciensis* and *V. anguillarum* (T6, □). Unexposed larvae and larvae exposed to single bacterial strains acted as controls: Negative Control (T1, ▪), only *V. anguillarum* (T2, ▴), only *P. gallaeciensis* wild type (T3, ▾), and only *P. gallaeciensis* TDA-negative mutant (T4, ♦).

Six days after the arrival of the embryos and inoculation, more than 50% of the larvae had hatched. Total cumulative hatching success was 79.2% (first trial 79.6% and second trial 78.7%). The initial mortality was lower in the first trial (16.6%) than in the second (24.8%). In the non-challenged and non-treated control, 34.7%±9.8% (average ± standard deviation) of the larvae had died by day 1, yet after the initial mortality only 2.8%±0% of the larvae died between day 2 and day 10, reaching an accumulated mortality of 37.5%±9.8% at day 10. The larvae challenged with *V. anguillarum* HI610 died rapidly and reached 100%±0% accumulated mortality. Treating *Vibrio*-challenged larvae with wild type *P. gallaeciensis* caused a significant reduction in accumulated mortality by day 10 to 12.5%±2.0% which was not only lower than in the challenged larvae but also lower than in the non-challenged (37.5%). 96.1%±1.1% of the hatched larvae that had received wild type *P. gallaeciensis* survived until day 10, when starvation set in (Figure 7, Figure S5). The larvae exposed to only *P. gallaeciensis* wild type or mutant had a cumulative mortality of 12.1%±3.1% at day 10. The TDA-negative mutant of *P. gallaeciensis* did reduce accumulated mortality of the challenged larvae to 68.8%±30.4% (Figure 7, Figure S5), but was not nearly as efficient as the TDA-producing wild type.

## Discussion

The present study demonstrates that *Phaeobacter gallaeciensis* is harmless and beneficial for the early life stages of cod. Equally important, *P. gallaeciensis* is highly efficient at preventing infections with *V. anguillarum*, and this probiotic effect can be achieved at the low temperature (7°C) used for the cod embryos and yolk sac larvae. It has previously been demonstrated that a *Phaeobacter* sp. can protect turbot larvae against vibriosis at higher temperatures (18°C) [29]. Non-infected larvae showed some level of initial mortality, which may have been due to opportunistic bacteria introduced with the embryos. Both challenged and unchallenged cod larvae exposed to *P. gallaeciensis* had a significantly lower initial mortality, indicating that the inherently occurring microbiota of the chorion may be controlled by the probiont.

A key question in the use of probiotics in aquaculture is how and where the probiont should be introduced to the system. Several studies have emphasized the potential role of feed organisms as a vehicle for probiotic bacteria [29], [48]–[51], or the potential of probiotic bacteria to control pathogenic bacteria in the feed cultures [51]–[53]. The majority of studies have focused on intestinal probiotic bacteria, and aimed at health-promoting effects within either the reared animal or the feed organism. In contrast, the present study takes a systems approach to preventing bacterial disease in aquaculture organisms, aiming at microbial control throughout the environment of the reared organism and the lower trophic levels of the production. Here it was found that cultures of two aquaculture-relevant algae and of the rotifer *B. plicatilis* can be colonized by *P. gallaeciensis* without compromising their growth, and that *P. gallaeciensis* in these cultures will strongly reduce, or eliminate fish-pathogenic *V. anguillarum*. Introducing *P. gallaeciensis* at this trophic level is very promising, since live feed is a common source of opportunistic pathogens [1], [5], [10], [11]. These findings corroborate the hypothesis from a previous study, that algae and rotifers in aquaculture can be cultured together with probiotic *Roseobacters*, and thus prevent proliferation of pathogens [22]. A reduction of a pathogenic *Vibrio* sp. by 3 log units, as it was achieved in the present study, is very promising in terms of larval health promotion, as only a one log reduction of the bacterial load in rotifers through UV radiation resulted in higher survival of turbot larvae [54]. Using probiotic bacteria, as compared to UV treatment, offers the advantage that nutrients are consumed, niches are occupied, and rapid re-growth of pathogens is prevented. It should be noted that the present study was done using gnothobiotic systems to rule out the influence of the inherent microbiota of algae and rotifer cultures. Thus, it cannot be determined, if or to what extent *P. gallaeciensis* would affect the inherent microbial communities of algae and rotifer cultures.

The inhibition of *V. anguillarum* by a *Phaeobacter* sp. in a model aquaculture setting has been studied once before: Planas *et al*. [29] demonstrated that mortality in turbot larvae infected with *V. anguillarum* could be reduced by *Phaeobacter* sp. 27-4. A duplicate tank setup was used, and both the pathogen and the probiont were enclosed in rotifers and fed to the larvae. In spite of delivery with the feed, the probiont was only found in the lumen of the larval gut and did not colonize the intestinal epithelium. In contrast to this, the present study did not aim at a probiotic effect in the intestinal tract of the larvae, but assesses the potential of *Phaeobacter* to eliminate the pathogen in the environment of the larvae or embryos. It should be mentioned that, as larvae start to drink shortly after hatching [55], an intestinal presence of pathogens and probionts can occur. *Phaeobacter* sp. 27-4 is a TDA-producer, however, as opposed to *P. gallaeciensis* BS107, it produces TDA only in stagnant culture [26], suggesting that its TDA production may be more delimited and that BS107 could be more antagonistic *in vivo*.

In the challenge trial the TDA-negative mutant reduced the initial mortality as efficiently as the wild type, but could not prevent infection in the majority of the larvae. The probiotic effect of the mutant could be explained by competition for nutrients, space, or other resources or it could be attributed to a direct immunostimulatory effect on the larvae [56]–[59]. The mutated gene that renders *P. gallaeciensis* BS107-Pda8 unable to produce TDA belongs to an operon encoding the parts of a transport protein, which has not yet been reported to be involved in TDA production [42]. The role of this transmembrane protein in TDA production has not been investigated. It cannot be excluded that this mutation has pleiotropic phenotypic effects, and other functions than TDA production might be affected and could affect the antagonistic properties of *Phaeobacter gallaeciensis*. Nonetheless, the experiment using pure TDA indicated that this compound indeed has a major inhibitory effect against *V. anguillarum* in the algal system.

The difference in *Vibrio*-antagonism between the TDA-negative mutant and the wild type suggested that TDA production is the trait that enables *P. gallaeciensis* to antagonize *V. anguillarum*. However, TDA was not detectable by chemical analysis of the *Phaeobacter-Tetraselmis* co-cultures. Since a *tdaC*-promoter fusion (*tdaCp*::*gfp*), demonstrated that *tdaC* is expressed by *P. gallaeciensis* in particles in the algae cultures, the reason for the lack of chemical detection could be that the TDA concentration only reaches inhibitory concentrations in *Phaeobacter*-colonized particles. TDA is likely concentrated within and around the particles, adhering to organic mass of the particle, or being kept within the EPS produced by *P. gallaeciensis*. From an ecological point of view, for a particle-associated marine bacterium the production of an antagonistic compound would be more efficient if the compound was not dispersed, but kept in the close vicinity to fend off possible competitors.

Although TDA-producing *Phaeobacter* and *Ruegeria* spp. are likely to be already present in larviculture systems, their antagonistic properties, which may depend on growth conditions, are probably different from *P. gallaeciensis* BS107 [22], [26]. A preliminary experiment to this study showed that only a few of the tested *Phaeobacter* and *Ruegeria* strains were antagonistic in *T. suecica* cultures, whereas all of them did account for large inhibition zones in agar-based assays. Therefore, introduction of *P. gallaeciensis* BS107 in algae and rotifer cultures would likely enhance larval survival even though other *Roseobacters* are already present in the system. Its growth-promoting effect on rotifers may offer an unexpected additional advantage. Whether that is due to the nutritive value of the bacteria or to a potential role in the rotifer gut is not known. In the present study rotifer growth was not adversely affected by *V. anguillarum*. Nevertheless, a *V. anguillarum* strain was reported to cause pronounced growth inhibition of rotifers under suboptimal feeding schemes [60], which could possibly be remediated by *P. gallaeciensis*.

It cannot be predicted, if and how other pathogens in algae and rotifer cultures would be suppressed by *P. gallaeciensis*, however a range of fish pathogens are inhibited *in vitro* by *P. gallaeciensis* [22], [25] indicating that it likely could protect against other pathogens than *V. anguillarum*. Porsby *et al*. [61] have addressed the concern that resistance to TDA could develop, and found, using several experimental approaches, that no resistant mutants or variants could be isolated, neither from short-term selection cultures containing different concentrations of TDA nor from long-term adaptation cultures (>300 generations) containing increasing concentrations of TDA.

A recent study demonstrated that *P. gallaeciensis*, when incubated with p-coumaric acid, produced potent algicides, the roseobactins, which were effective against different microalgae, among them *T. suecica* [62]. P-coumaric acid is a degradation product of lignin, which is contained not only in terrestrial plants, but also in algae. Production of the algicides was only possible in concentrations of p-coumaric acid above 0.4 mM. The authors hypothesized that *P. gallaeciensis* contributes to algal health and growth by secreting TDA and phenylacetic acid, but will produce algicides in presence of p-coumarate, which is an indicator for algal senescence, in order to utilize the algal biomass for its own growth [62]. However, in the present study no negative effect of *P. gallaeciensis* on algal growth was observed. Possibly the levels of p-coumaric acid in the cultures of microalgae were too low for roseobacticide production. The environmental ecological niche of *P. gallaeciensis* has not yet been described, although studies of Rao *et al*. [63]–[65] indicate its preference for the surface of macroalgae, which during their decay may account for higher local concentrations of p-coumaric acid than microalgae. In an aquaculture farm, *Phaeobacter* spp. have been found to “naturally” occur on solid surfaces, whereas only *Ruegeria* spp. were inherently associated with algae cultures [26].

Based on the present findings, it is hypothesized that *P. gallaeciensis* can be used in marine larviculture, as a means of controlling the ambient, potentially harmful microbiota in cultures of rotifers and microalgae, and as a prophylaxis against vibriosis in fish larvae.

## Supporting Information

Figure S1 **Concentrations of** ***Nannochloropsis oculata*** **and** ***Phaeobacter gallaeciensis*** **in the co-cultures.** Colony-forming units of *P. gallaeciensis* wild type (•) and the TDA-negative mutant (□), and concentrations of *N. oculata* with *V. anguillarum* (▴), *N. oculata* with *P. gallaeciensis* wild type (▾), *N. oculata* with *P. gallaeciensis* TDA-negative mutant (♦), and axenic *N. oculata* (▪) in the dense (A) and less dense (B) cultures.
(TIF)

Figure S2 **Reduction of** ***Vibrio anguillarum*** **by** ***Phaeobacter gallaeciensis*** **in cultures of** ***Tetraselmis suecica*** **.** Colony-forming units of *V. anguillarum* inoculated at 10^2^ cfu/ml (A) and at 10^3^ cfu/ml (B) in presence of *P. gallaeciensis* wild type (▪), in presence of the *P. gallaeciensis* TDA-negative mutant (▴), and in the monoxenic control (▾).
(TIF)

Figure S3 **Reduction of** ***Vibrio anguillarum*** **by** ***Phaeobacter gallaeciensis*** **in cultures of** ***Nannochloropsis oculata*** **.** Colony-forming units of *V. anguillarum* in presence of *P. gallaeciensis* wild type (▴), in presence of the *P. gallaeciensis* TDA-negative mutant (▾), and in the monoxenic control (▪), in dense (3×10^7^ cells/ml; A) and less dense (1–7×10^6^ cells/ml; B) cultures of *N. oculata*.
(TIF)

Figure S4 **Influence of bacterial strains on rotifer growth.** Rotifer numbers in co-culture with *P. gallaeciensis* wild type (▾), with the TDA-negative mutant of *P. gallaeciensis* (♦), with only *V. anguillarum* (▴), and axenic rotifers (▪), second experiment. All bacteria were inoculated at day 0. Both *P. gallaeciensis* strains promoted rotifer growth, whereas *V. anguillarum* had no influence.
(TIF)

Figure S5 **Mortality of cod larvae during the challenge trials.** Mean values of two independent triplicate experiments with error bars indicating standard deviations. The single-larvae cultures were simultaneously inoculated with *P. gallaeciensis* wild type and *V. anguillarum* (T5, •), or with the TDA-negative mutant of *P. gallaeciensis* and *V. anguillarum* (T6, □). Unexposed larvae and larvae exposed to single bacterial strains acted as controls: Negative Control (T1, ▪), only *V. anguillarum* (T2, ▴), only *P. gallaeciensis* wild type (T3, ▾), and only *P. gallaeciensis* TDA-negative mutant (T4, ♦).
(TIF)
