# Supplementary figures and images for: Phaeobacter gallaeciensis Reduces Vibrio anguillarum in Cultures of Microalgae and Rotifers, and Prevents Vibriosis in Cod Larvae
Source: PLoS One. 2012 Aug 22;7(8):e43996. doi: 10.1371/journal.pone.0043996 (PMC3425499; doi:10.1371/journal.pone.0043996)

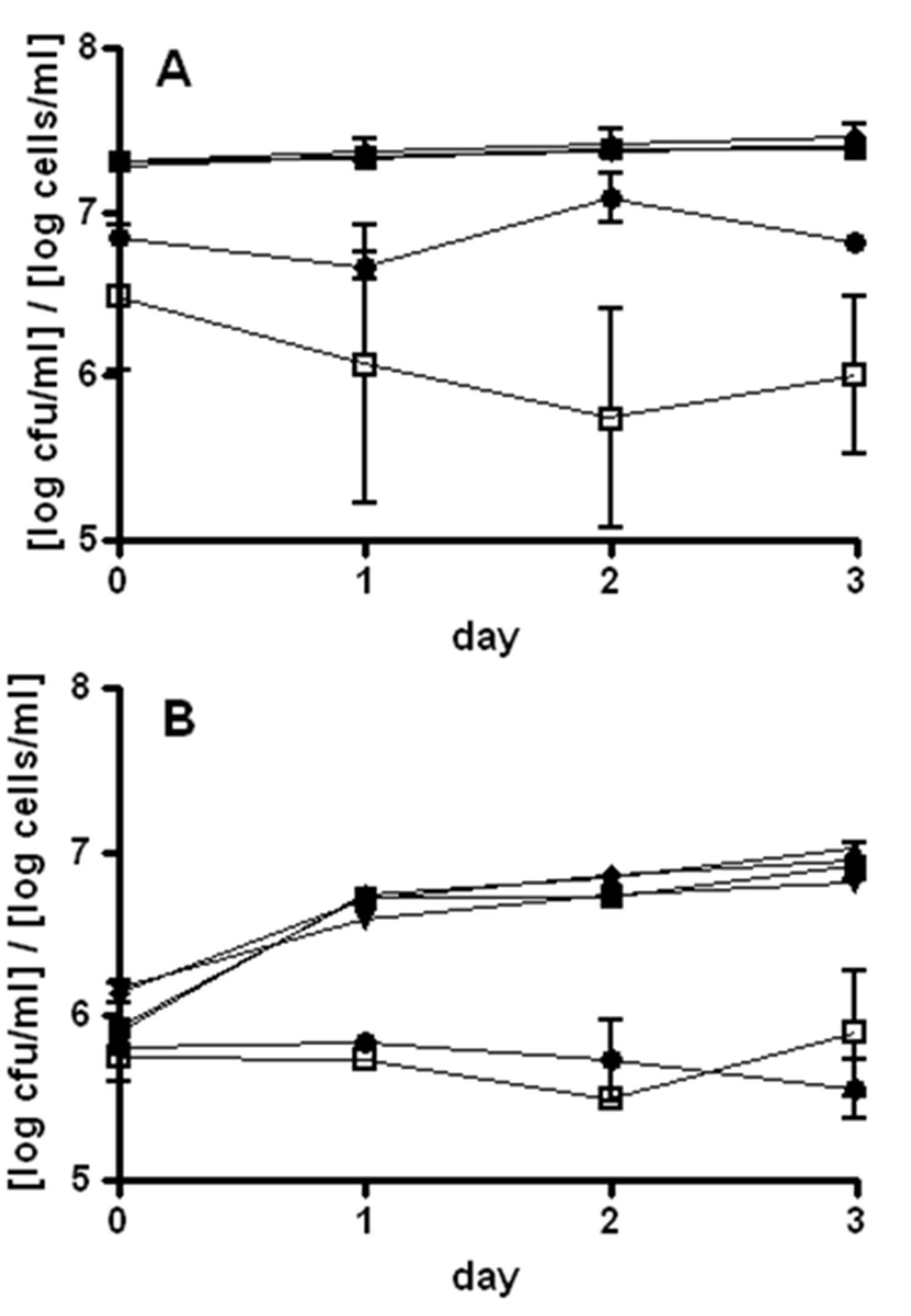

Supplement: Figure S1 — Concentrations of Nannochloropsis oculata and Phaeobacter gallaeciensis in the co-cultures. Colony-forming units of P. gallaeciensis wild type (•) and the TDA-negative mutant (□), and concentrations of N. oculata with V. anguillarum (▴), N. oculata with P. gallaeciensis wild type (▾), N. oculata with P. gallaeciensis TDA-negative mutant (♦), and axenic N. oculata (▪) in the dense (A) and less dense (B) cultures. (TIF) [file pone.0043996.s001.tif]

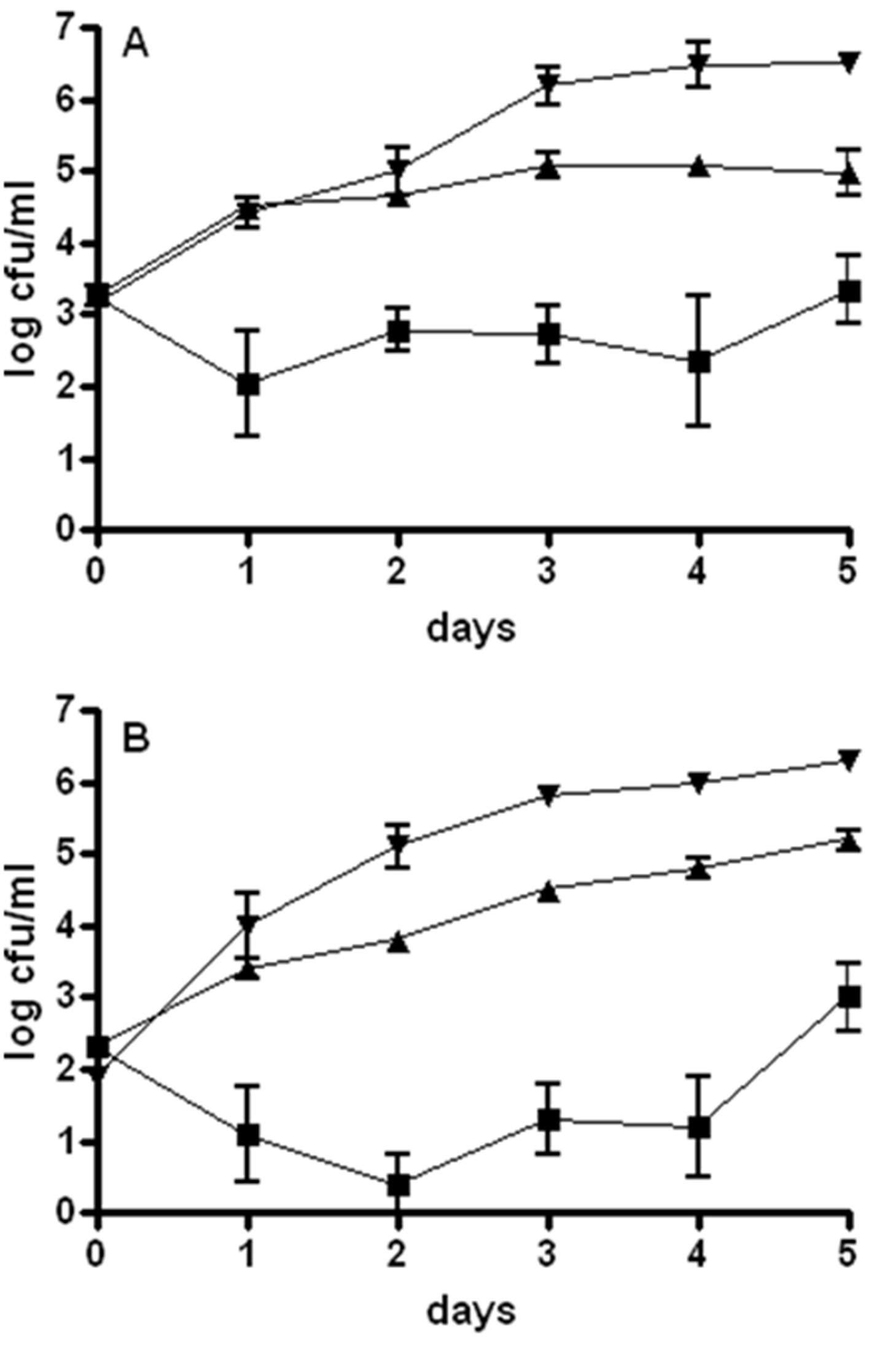

Supplement: Figure S2 — Reduction of Vibrio anguillarum by Phaeobacter gallaeciensis in cultures of Tetraselmis suecica . Colony-forming units of V. anguillarum inoculated at 102 cfu/ml (A) and at 103 cfu/ml (B) in presence of P. gallaeciensis wild type (▪), in presence of the P. gallaeciensis TDA-negative mutant (▴), and in the monoxenic control (▾). (TIF) [file pone.0043996.s002.tif]

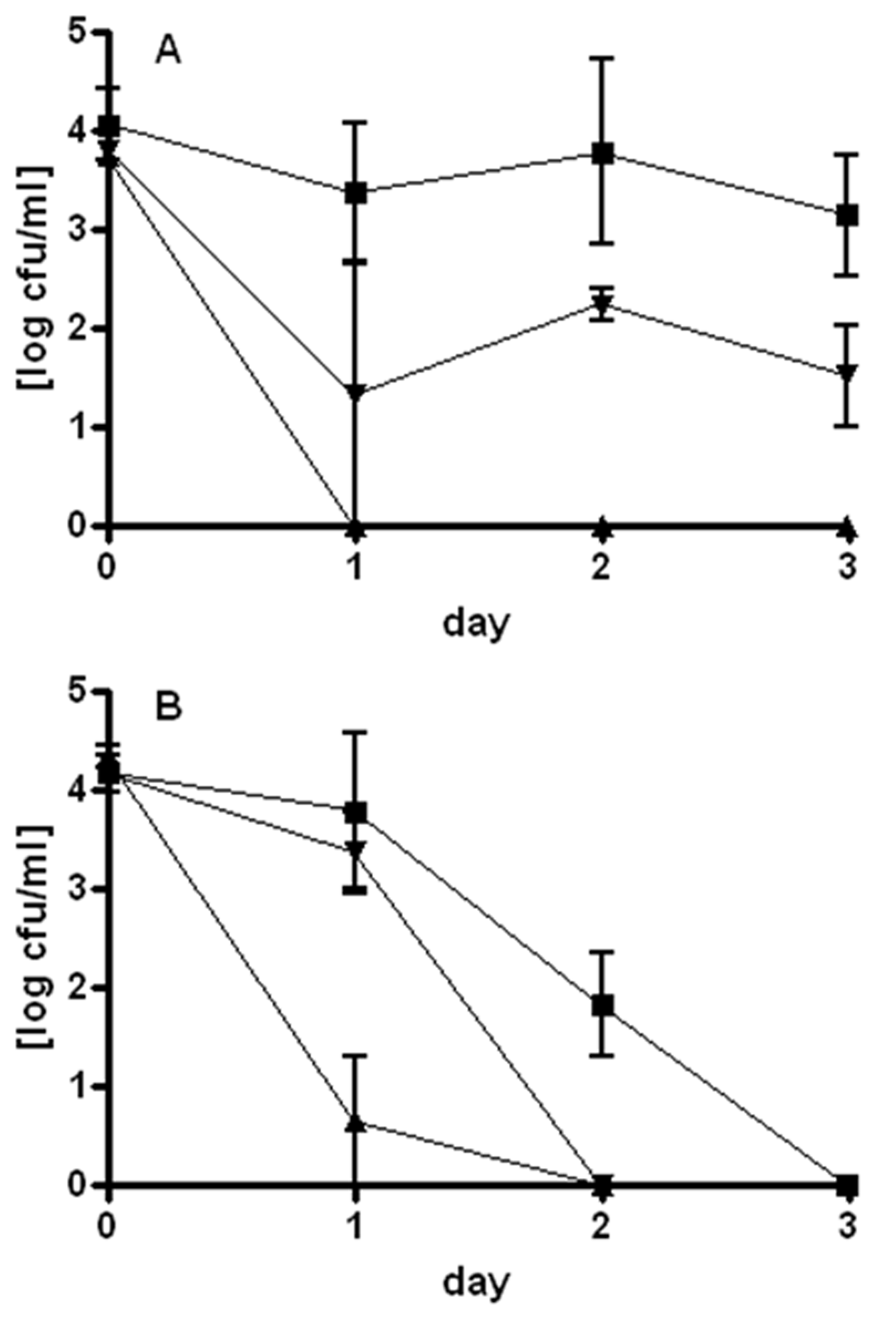

Supplement: Figure S3 — Reduction of Vibrio anguillarum by Phaeobacter gallaeciensis in cultures of Nannochloropsis oculata . Colony-forming units of V. anguillarum in presence of P. gallaeciensis wild type (▴), in presence of the P. gallaeciensis TDA-negative mutant (▾), and in the monoxenic control (▪), in dense (3×10^7^ cells/ml; A) and less dense (1–7×10^6^ cells/ml; B) cultures of N. oculata. (TIF) [file pone.0043996.s003.tif]

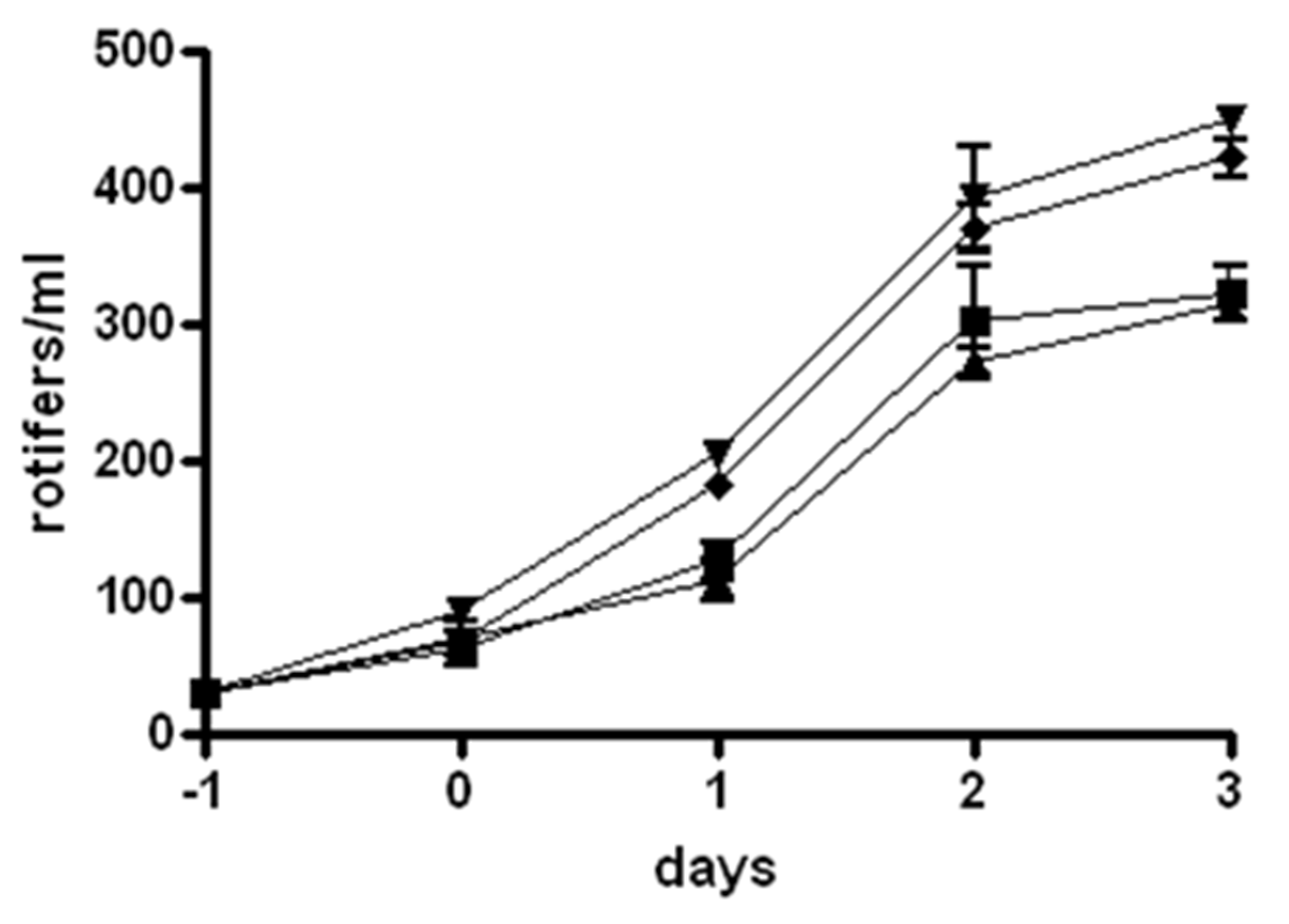

Supplement: Figure S4 — Influence of bacterial strains on rotifer growth. Rotifer numbers in co-culture with P. gallaeciensis wild type (▾), with the TDA-negative mutant of P. gallaeciensis (♦), with only V. anguillarum (▴), and axenic rotifers (▪), second experiment. All bacteria were inoculated at day 0. Both P. gallaeciensis strains promoted rotifer growth, whereas V. anguillarum had no influence. (TIF) [file pone.0043996.s004.tif]

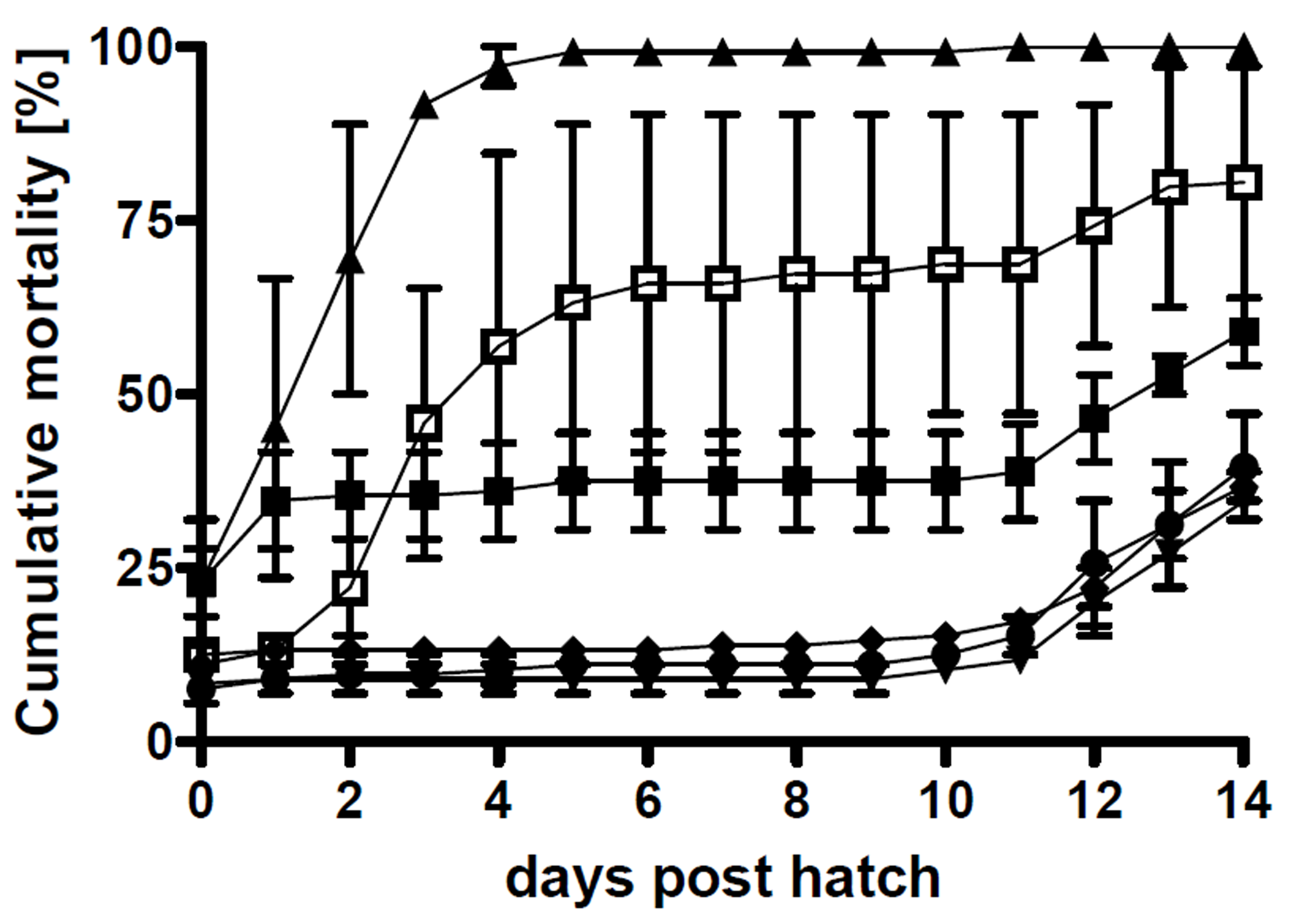

Supplement: Figure S5 — Mortality of cod larvae during the challenge trials. Mean values of two independent triplicate experiments with error bars indicating standard deviations. The single-larvae cultures were simultaneously inoculated with P. gallaeciensis wild type and V. anguillarum (T5, •), or with the TDA-negative mutant of P. gallaeciensis and V. anguillarum (T6, □). Unexposed larvae and larvae exposed to single bacterial strains acted as controls: Negative Control (T1, ▪), only V. anguillarum (T2, ▴), only P. gallaeciensis wild type (T3, ▾), and only P. gallaeciensis TDA-negative mutant (T4, ♦). (TIF) [file pone.0043996.s005.tif]
